# Post-phacoemulsification iris changes in eyes with glaucoma or glaucoma suspect status

**DOI:** 10.1371/journal.pone.0208776

**Published:** 2018-12-13

**Authors:** Qinyun Wang, Claudio I. Perez, Marissé Masis, Max Feinstein, Marta Mora, Shan C. Lin, Yen C. Hsia

**Affiliations:** 1 University of California at San Francisco, Department of Ophthalmology, San Francisco, California, United States of America; 2 Fundación Oftalmológica los Andes, Universidad los Andes, Santiago, Chile; Edith Wolfson Medical Center, ISRAEL

## Abstract

**Purpose:**

This prospective study used anterior segment optical coherence tomography (AS-OCT) to determine how phacoemulsification (phaco) changes iris parameters in eyes with glaucoma or glaucoma suspect status.

**Methods:**

Using Visante AS-OCT (Carl Zeiss Meditec AG), the following pre- and post-phaco parameters were measured: IT750 = iris thickness at 750 μm from the scleral spur; IT2000 = iris thickness 2000 μm from the scleral spur; ITCM = the maximum iris thickness at the middle one third of the iris; ICURV = iris curvature; IAREA = iris area; and pupil size = pupil diameter (mm). Only high-quality images with an identifiable scleral spur were included, and only the nasal quadrant was analyzed. A single glaucoma specialist analyzed the parameters according to the Zhongshan Angle Assessment Program (ZAAP, Guangzhou, China). Multivariate analysis was performed using mixed effects regression correcting for age, gender, and ethnicity.

**Results:**

89 subjects and 110 eyes were included in this study. The mean age of subjects was 74.83 {+/-} 8.69 years old. Most common diagnoses were POAG and glaucoma suspect (23% and 52%, respectively), and 16% of subjects had an LPI. In multivariate analysis of AS-OCT parameters, decreases in IT750, IT2000, ITCM, ICURV, and pupil size were statistically significant (p<0.05).

**Conclusions:**

After phacoemulsification, eyes with glaucoma as well as glaucoma suspect eyes have thinner irises and smaller pupils. This may lead to less iris-mediated aqueous outflow obstruction, providing support for early phacoemulsification glaucoma treatment.

**Translational relevance:**

Our AS-OCT imaging findings may guide clinical practice as iris parameters become increasingly relevant in preoperative phaco planning.

## Introduction

Glaucoma and cataract, two well-known disease entities with an estimated comorbidity of 19.1%, are the leading causes of blindness worldwide [1–3]. Currently, the only modifiable glaucoma risk factor is intraocular pressure (IOP), which is often but not always elevated in glaucoma patients [4]. Additionally, in patients with primary glaucoma, specific treatment regimens and prognoses depend not only on IOP, but also on anterior chamber angle morphology [4,5]. Existing literature demonstrates that cataract surgery, one of the most commonly performed surgical procedures around the world, tends to lower IOP as well as favorably modify anterior chamber parameters such as lens vault and iris thickness [5–10].

The IOP-lowering and anterior chamber-altering effect of phacoemulsification cataract surgery (phaco) has been studied in both nonglaucomatous and glaucomatous eyes [9, 11–31]. Given the improved safety profile of modern phaco compared to traditional incisional glaucoma surgeries, phaco has been advocated as an early means of lowering IOP [7,32]. Specifically, patients with primary angle closure (PAC) and primary angle closure glaucoma (PACG) derive greater benefit from clear lens extraction compared to laser peripheral iridotomy [23,33,34]. In OAG, studies have also demonstrated post-operative IOP reduction [12–14] and significant post-operative changes in anterior segment parameters such as angle-opening distance (AOD500), supporting the theory that anatomical changes in the anterior segment may be responsible for the IOP-lowering effects of phacoemulsification [7,12].

Anterior segment optical coherence tomography (AS-OCT) allows quantification of various anterior segment parameters and has provided valuable information regarding pre- and post-phaco changes in the anterior segment [35]. Previously studied AS-OCT parameters include: anterior chamber volume (ACV), anterior chamber width (ACW), iris area, and iris curvature (I-curve) [12,14]. It is now well-established that phaco deepens the anterior chamber and widens the angle [6,12,14,36–38], but there is less dedicated research into how this surgery impacts the iris itself [7,12].

The iris is a permeable, sponge-like structure whose cross-sectional area and volume vary under certain conditions [39,40]. The spongy quality of the iris stroma may exert significant influence on anterior chamber anatomy [40]. While phaco demonstrably alters the anterior chamber angle and other parameters, the impact of phaco on the iris is unknown. As such, we conducted a prospective study evaluating the effect of phaco on AS-OCT iris parameters in glaucoma and glaucoma suspect patients.

## Patients and methods

This was a prospective study approved by the institutional review board (IRB) at the University of California, San Francisco (UCSF), California, USA. Written informed consent was obtained from all patients prior to enrollment. Patients were consecutively recruited from the UCSF glaucoma clinics from May 2012 through March 2017.

### Inclusion criteria

Inclusion criteria included age older than 18 years, presence of visually significant cataract(s) that were removed via uncomplicated phacoemulsification, and completion of at least 1 pre-operative and 1 post-operative AS-OCT imaging session. Both glaucoma and glaucoma suspect eyes were included; eyes were determined to have a diagnosis of glaucoma as defined by 1) the use of glaucoma medications plus 2) the presence of glaucomatous disc excavation and glaucomatous visual field defects, OR glaucomatous disc cupping of 0.9 or greater in patients unable to undergo visual field testing. Glaucoma suspects were defined as eyes with glaucomatous disc excavation without glaucomatous visual field defects. Both eyes of patients were included in the study if they met inclusion criteria.

### Exclusion criteria

Exclusion criteria included age less than 18 years; phaco with complicated intraoperative (e.g., vitreous loss, need for Malyugin ring) and/or postoperative course; prior history of intraocular surgery (e.g. iStent, trabeculectomy); other ocular conditions affecting visual acuity and/or IOP; ocular trauma; evidence of pigment dispersion or pseudoexfoliation; peripheral anterior synechiae (PAS) seen on gonioscopy; and/or issues with AS-OCT image analysis. Eyes that have undergone laser peripheral iridotomy (LPI) or laser trabeculoplasty were not excluded.

Demographic information, preoperative assessment, and post-operative values from 176 patients and 240 eyes were initially obtained from our database of subjects who enrolled to be followed with anterior segment imaging. Ultimately, 87 subjects and 130 eyes were excluded based on the exclusion criteria above. Clinical data of interest included glaucoma diagnosis and IOP. Of note, IOP was measured in the same manner (e.g., undilated eye, Goldmann applanation tonometry), by the same clinician (S.C.L), during the same time of day (between 1PM and 4PM). The IOP was checked twice and mean IOP was used in this study. If the IOP measurement from the 2 tests differed by more than 2mmHg, a third value was obtained and the median was chosen.

### Surgical technique

Phacoemulsification was performed by the same surgeon (S.C.L) using standard techniques. A temporal clear corneal incision was used, and a single piece acrylic intraocular lens (IOL) (Acrysof SN60WF, Alcon Laboratories, Inc., Fort Worth, TX) was implanted in all operative eyes. The standard postoperative eyedrop regimen included topical antibiotics 4 times daily for 1 week, prednisolone acetate 1.0% 4 times daily with a weekly taper, and ketorolac tromethamine 0.5% 4 times daily with a weekly taper.

### Postoperative follow-up

Postoperative follow-up visits were performed at 3 months, 6 months, and 1 year after surgery. Each visit included visual acuity testing, IOP measurement, a complete slit lamp examination, and AS-OCT imaging.

### Anterior segment optical coherence tomography (AS-OCT) and definition of parameters

Pre- and post-operative AS-OCT data were gathered using a Visante AS-OCT device (Carl Zeiss Meditec AG, Dublin, CA). Details of the imaging procedures and analysis have been previously described [14]. The following iris parameters were measured at baseline and at post-operative month 3 and beyond: IT750 = iris thickness measured at 750 μm from the scleral spur; IT2000 = iris thickness 2000 μm from the scleral spur; ITCM = the maximum iris thickness at the middle one third of the iris; ICURV = iris curvature; and IAREA = iris area. Pupil size, or pupil diameter in mm, was also evaluated. Only high-quality images with an identifiable scleral spur were included, and only the nasal quadrant was analyzed, as the other quadrants may have been distorted by eyelid manipulation or phacoemulsification trauma. Of note, only images obtained in the dark were used in this study.

A single glaucoma specialist analyzed the AS-OCT parameters according to established protocols using the Zhongshan Angle Assessment Program (ZAAP, Guangzhou, China). Image analysis was performed on post-operative AS-OCT images taken anywhere from 3 months to 1 year after the initial surgery; these different end points were due to inconsistent image availability arising from ZAAP segmentation issues encountered during analysis. Of note, current literature suggests that anterior segment parameters do not undergo much change beyond post-operative month 3 [14]. Therefore, all reported post-operative measurements taken after post-operative month 3 were treated equivalently. Multivariate analysis was subsequently performed using mixed effects regression correcting for age, gender, and ethnicity.

### Statistical analysis

The Student’s *t* test was used to calculate statistical differences between paired continuous variables. Categorical variables were compared using the chi-square test. Linear mixed-effects regression analysis was used to assess the correlation between AS-OCT parameters, change in AS-OCT parameters (pre-operative values [in mm]–post-operative values), change in IOP (postoperative IOP minus preoperative IOP), and presence of LPI in univariate analysis and multivariate analysis. A *P* value less than 0.05 was considered statistically significant.

## Results

This study enrolled a total of 176 subjects and 240 eyes; 89 subjects and 110 eyes were included for analysis after study exclusion criteria were applied. Data were also excluded if ZAAP segmentation issues (e.g., negative iris values, n = 57) were encountered during analysis. Of included subjects, 63 were women and 47 were men. Subjects had a range of glaucoma-related diagnoses as outlined in Table 1. The majority of subjects carried a diagnosis of POAG and glaucoma suspect (23% and 52%, respectively), and 16% of subjects had an LPI performed in one or both eyes previously.

**Table 1:** Baseline patient demographics.

Parameter		Subjects (n)	% Total
**Age**	Mean ± SD: 74.83 ± 8.69 Age Range: 55, 97		
**Sex**	Female	63	57.27
	Male	47	42.73
**Race**	Asian	51	46.36
	Caucasian	34	30.91
	Hispanic	12	10.91
	Other	13	11.82
Glaucoma Diagnosis*	NTG	3	3.37
	PACG	3	3.37
	PACG + MMG	1	1.12
	PACS	1	1.12
	POAG	23	25.84
	Suspect	52	58.43
	Other	6	6.74
**Previous LPI**		18	16.36

*NTG = Normal Tension Glaucoma; PACG = Primary Angle Closure Glaucoma; PACG + MMG = Primary Angle Closure Glaucoma + Mixed Mechanism Glaucoma; PACS = Primary Angle Closure Suspect; POAG = Primary Open Angle Glaucoma

Table 2 outlines pre- and post-operative IOP data and AS-OCT parameters in all 110 eyes. Post-operative AS-OCT parameters, including pupil size, were obtained up to 1 year after uncomplicated phaco. These values were noted to be uniformly lower than pre-operative values in eyes with glaucoma or glaucoma suspect status. Univariate analysis (Table 3) found this pattern to be statistically significant for IT750, IT2000, ITCM, pupil size (p<0.005 for all 4), and ICURV (p = 0.04); after adjusting for age, gender, and ethnicity, the change between pre- and post-operative IT750, IT2000, ITCM, and pupil size was still statistically significant (p<0.005).

**Table 2:** Pre and post-operative IOP and anterior segment-OCT (AS-OCT) parameters†.

Parameter	Pre-Op (mean ± SD)	Post-Op* (mean ± SD)
**IOP**	15.3±3.31	12.52±2.53
**IT750 (mm)**	0.47±0.16	0.45±0.12
**IT2000 (mm)**	0.49±0.19	0.45±0.08
**ITCM (mm)**	0.63±0.23	0.61±0.19
**IAREA (mm**^**2**^**)**	1.62±0.53	1.38±0.48
**ICURV (mm)**	0.30±0.18	0.16±0.08
**Pupil Size (mm)**	4.19±0.85	3.89±0.84

Iris parameter definitions: IT750 = iris thickness measured at 750 μm from the scleral spur; IT2000 = iris thickness 2000 μm from the scleral spur; ITCM = the maximum iris thickness at the middle one third of the iris; ICURV = iris curvature; IAREA = iris area. Pupil size = pupil diameter.

†In dark conditions.

*Post-op includes data ranging from 3 months to 1 year after surgery.

**Table 3:** Univariate and multivariate analysis* of the association between pre and post-operative AS-OCT Iris parameters.

	Univariate Analysis				Multivariate Analysis*			
Parameter	β	95% CI	p-value	SD	β	95% CI	p-value	SD
**IT750 (mm)**	0.40	0.16, 0.64	<0.005	0.12	0.30	0.07, 0.52	<0.005	0.11
**IT2000 (mm)**	1.05	0.62, 1.47	<0.005	0.22	0.46	0.04, 0.88	0.032	0.21
**ITCM (mm)**	0.63	0.43, 0.83	<0.005	0.10	0.59	0.39, 0.80	<0.005	0.11
**IAREA (mm**^**2**^**)**	0.04	-0.17, 0.25	0.711	0.11	-0.01	-0.21, 0.18	0.896	0.10
**ICURV (mm)**	0.42	0.02, 0.81	0.040	0.20	0.38	-0.03, 0.80	0.066	0.21
**Pupil Size** **(mm)**	0.61	0.47, 0.76	<0.005	0.76	0.62	0.47, 0.76	<0.005	0.75

*multivariate analysis was performed using mixed effects regression correcting for age, gender and ethnicity

Of note, in Table 4, pupil size and presence of an LPI were not found to impact pre-and post-operative iris parameters. While the association between pre- and post-operative IT2000 (p = 0.018) and ICURV (p = 0.005) were statistically significantly affected by an LPI, the β coefficients for these two parameters were extremely low (0.13, and -0.13, respectively).

**Table 4:** Multivariate* analysis of the association between Iris parameters, pupil size, and LPI.

	Pre-Op Pupil Size				Post-Op Pupil Size				LPI			
Parameter	β	95% CI	p-value	SD	β	95% CI	p-value	SD	β	95% CI	p-value	SD
**IT750 (mm)**	0.30	-0.01, 0.06	0.106	0.02	0.02	0.00, 0.05	0.113	0.01	0.06	-0.15, 0.52	0.267	0.17
**IT2000 (mm)**	0.02	-0.16, 0.05	0.310	0.02	0.02	0.00, 0.03	0.024	0.01	0.13	0.02, 0.24	0.018	0.06
**ITCM (mm)**	0.06	0.14, 0.10	0.011	0.02	0.07	0.04, 0.10	<0.005	0.02	0.12	0.00, 0.24	0.050	0.06
**IAREA (mm**^**2**^**)**	-0.03	0.16, 0.09	0.595	0.06	-0.02	-0.04, -0.01	0.012	0.01	0.25	-0.06, 0.56	0.120	0.16
**ICURV (mm)**	-0.07	-0.11, 0.03	<0.005	0.02	0.32	-2.57, 3.12	0.828	1.48	-0.13	-0.23, -0.04	0.005	0.05

*multivariate analysis was performed using mixed effects regression correcting for age, gender and ethnicity

Table 5 demonstrates that the post-phaco IOP change experienced by eyes with glaucoma or glaucoma suspect status is not statistically significantly associated with pre-operative AS-OCT iris parameters, post-operative AS-OCT iris parameters, and ΔIris (or, pre-operative minus post-operative iris values in mm).

**Table 5:** Multivariate analysis* of the association between AS-OCT Iris parameters and changes in IOP: Is IOP change after phacoemulsification related to pre, post, and changes in AS-OCT Iris parameter values?

	IOP change and Pre-Op Iris Values				IOP change and Post-Op Iris Values				IOP change and ΔIris Parameters (Pre-Post)			
Parameter	β	95% CI	p-value	SD	β	95% CI	p-value	SD	β	95% CI	p-value	SD
**IT750 (mm)**	0.30	-2.60, 3.18	0.843	1.47	-1.16	-5.25, 2.93	0.579	2.09	0.53	-2.10, 3.16	0.694	1.34
**IT2000 (mm)**	1.33	-1.06, 3.71	0.275	1.22	-0.51	-7.32, 6.30	0.884	1.22	1.32	-1.01, 3.70	0.279	1.22
**ITCM (mm)**	1.38	-0.77, 3.53	0.207	1.10	-0.37	-2.97, 2.22	0.778	1.33	1.07	-0.95, 3.10	0.300	1.03
**IAREA (mm**^**2**^**)**	0.03	-0.80, 0.85	0.949	0.42	-0.85	-1.85, 0.14	0.093	0.51	0.35	-0.27, 0.97	0.262	0.32
**ICURV (mm)**	-1.63	-4.10, 0.81	0.190	1.24	-5.87	-11.73, -0.01	0.050	2.99	-0.95	-3.37, 1.47	0.443	1.24

ΔIris = pre-op minus post-op iris parameter values

## Discussion

The iris is a dynamic, light-responsive organ comprised of the following structures from anterior to posterior: a surface layer of irregular cells, a fibrovascular stroma, the dilator and sphincter muscles, and a two-cell layer thick iris pigment epithelium (IPE) [41]. It is highly permeable to even macroscopic particles, and, being fully immersed in aqueous humor, allows the free passage of fluid between stroma and aqueous [40,41]. This fluid exchange is best demonstrated by the observation that a normal iris routinely loses area and volume after pupil dilation both in dark and pharmacologic conditions [40,42–44].

Such changes are thought to be due to movement of extracellular water in and out of the loose connective tissue of the iris stroma, demonstrating Quigley’s comparison of the iris to a sponge [39]. It is believed that the spongier an iris, the greater its volume loss during pupil dilation; presumably, greater volume loss prevents aqueous obstruction at the trabecular meshwork and potentially at the iris-lens channel, leaving an eye less vulnerable to increases in IOP [39,40,42–45].

There has been heightened interest in this area of iris anatomy, with recent research aimed at identifying surface iris landmarks that may accurately approximate iris sponginess. For instance, iris crypts and furrows, landmarks that are easily identified at the slip lamp, have been found to be potential surrogates for iris sponginess–irises with longer, more numerous furrows may lose less volume on pupil dilation; those with more crypts may lose more volume on pupil dilation [46–48]. In this study, AS-OCT, a reproducible and easy-to-learn imaging method, may offer yet another noninvasive method of directly assessing iris stromal characteristics. Potentially representative parameters include IT750, IT2000, and ITCM.

This study demonstrates that after phaco, eyes with either glaucoma or glaucoma suspect status undergo statistically significant decreases in iris thickness, specifically IT750, IT2000, and ITCM, in dark conditions (Table 3). There is also a significant decrease in pupil size after phaco. Of note, after pre- and post-op iris values were adjusted for pre- and post-op pupil size respectively, it was found that pupil size did not impact any of the iris parameters. Therefore, phacoemulsification may lead to thinner irises and less dark-adapted pupil dilation in eyes with glaucoma as well as eyes with glaucoma suspect status. The “dark” AS-OCT values included in this paper may need to be compared to future “light” values in order to definitely clarify phaco effects on dynamic iris parameters as well as iris sponginess.

It has previously been noted that the presence of a peripheral iridotomy does not change the degree of iris volume loss with dilation, suggesting that iris sponginess is an innate property that cannot be altered [38]. In this study, a subgroup analysis was performed on the 18 subjects with an LPI. As shown in Table 4, AS-OCT iris parameters of eyes with an LPI were not significantly impacted by the LPI (β coefficients ranged from 0.13, and -0.13) and therefore experienced the same degree of thickness loss and change in pupil size compared to irises without an LPI. In other words, LPI does not appear to alter the degree of phaco-induced iris change, at least under static, dark-only conditions.

Our study suggests that phacoemulsification thins the iris and leads to smaller pupil diameters up to a year after the initial surgery. Potential mechanisms of iris thinning during routine phacoemulsification include direct trauma from the phaco probe and indirect damage to iris structures from the cavitation energy generated by the phaco tip [49]. While the exact effects of mechanical phacoemulsification energy on iris stroma are unknown, existing literature suggests that on a histopathological level, phaco-generated ultrasonic waves induce intracytoplasmic vacuolation within the sphincter and dilator muscles of both human and simian irises [50,51]. Our data indicates that these post-op changes may decrease the extent of pupil dilation in the dark, pointing to reduced iris movement in dark conditions and therefore potentially reduced incidence of iris-trabecular meshwork apposition. It is difficult to draw definitive conclusions, however, without iris data gathered in photopic conditions; both lights on and lights off data will be needed to make conclusions about dynamic post-operative iris changes.

Iris parameters may be important data points in preoperative phacoemulsification planning. For example, in eyes with advanced cataract and therefore increased lens vault [12], the resultant anteriorization of the lens-iris diaphragm narrows the anterior chamber angle and obstructs aqueous outflow; there may also be concomitant narrowing of the iris-lens channel, creating additional obstruction to aqueous outflow at the level of the pupil and then subsequent the angle through pupillary block. It is possible that thinner irises are less prone to such obstructions and can help favorably modulate IOP. Therefore, preoperative measures such as IT750, IT2000, and ITCM may shed light on which glaucoma and glaucoma suspect patients may be more at risk for IOP spikes and therefore may benefit most from the iris-thinning effects of phacoemulsification.

Along this vein, the present study found that iris parameters were not associated with post-operative IOP changes. Post-phaco IOP reduction has previously been found to be a mean of 2.1 mmHg, without any change to glaucoma medication regimen [12]. Based on this information, this study may not have been powered appropriately to detect a statistically significant IOP change, for at least 100 subjects would be needed to yield an 80% power to detect a difference of 1.9 mmHg in IOP; unfortunately, while this study initially enrolled 176 subjects, it ultimately included only 89 subjects and 110 eyes. Future research along these lines should include more subjects as well as AS-OCT iris data from both light and dark conditions.

Using AS-OCT, our study demonstrates that iris thickness (as approximated by IT750, IT2000, and ITCM) may be altered by phacoemulsification in eyes with glaucoma as well as in eyes with glaucoma suspect status. However, these findings only apply to static data gathered in dark conditions. Our study has several limitations, including the small number of subjects, high exclusion rates (largely due to ZAAP segmentation issues), and the inclusion of iris parameters from dark conditions only. There are also very few subjects representing categories of glaucoma other than POAG. Additionally, since measurements of anterior segment parameters are partly dependent on manual identification of the scleral spurs, there is an element of human error and bias. We sought to minimize these factors through the use of a single grader of the AS-OCT images masked to the clinical results. Also, iris values (IT750, IT2000, IAREA, ICURV) represent focal anterior segment parameters and were not averaged over the temporal and nasal sectors in this report as has been done in some prior studies using AS-OCT. Due to concerns that temporal surgical sites may affect postoperative measurements, we utilized only the nasal segments of the AS-OCT images in our analyses. Finally, our post-operative AS-OCT images were obtained at different endpoints; while the current literature suggests that anterior segment parameters do not undergo much change beyond post-operative month 3, this may have still introduced some confounding elements.

Our findings not only highlight the importance of iris anatomy in early surgical glaucoma management, but also supports the potential role of AS-OCT imaging in current clinical glaucoma practice and future glaucoma research.

## Supporting information

S1 Data Limited data set containing pre and post-phaco iris values. (XLSX)
